# Narratives about distributed health literacy during the COVID-19 pandemic

**DOI:** 10.1177/13634593231215715

**Published:** 2023-12-14

**Authors:** Susana Silva, Helena Machado, Ilaria Galasso, Bettina M Zimmermann, Carlo Botrugno

**Affiliations:** Institute for Social Sciences, University of Minho, Portugal; Centre for Research in Anthropology (CRIA-UMinho/IN2PAST), Portugal; Institute for Social Sciences, University of Minho, Portugal; University College Dublin, Ireland; Institute of History and Ethics in Medicine, Department of Clinical Medicine, TUM School of Medicine and Health, Technical University of Munich, Germany; Institute for Biomedical Ethics, University of Basel, Switzerland; Institute of History and Ethics in Medicine, Department of Clinical Medicine, TUM School of Medicine and Health, Technical University of Munich, Germany; Institute of Philosophy & Multidisciplinary Center for Infectious Diseases, University of Bern, Switzerland; Research Unit on Everyday Bioethics and Ethics of Science, Department of Legal Sciences, University of Florence, Italy

**Keywords:** COVID-19, critical health literacy, distributed health literacy, literacy mediators

## Abstract

The promotion of health literacy was a key public health strategy during the COVID-19 pandemic. However, the role of social networks and relationships for support with health literacy-related tasks in the context of the COVID-19 pandemic is scarcely understood. Moving beyond traditional notions of health literacy, which focus on individual skills and knowledge, this study uses the concept of distributed health literacy to explore how individuals make meaning of and respond to health literacy and make their literacy skills available to others through their relational and socially situated and lived experiences of the COVID-19 pandemic. Drawing on 89 semi-structured interviews conducted in three European countries (Italy, Portugal, and Switzerland) between October and December 2021, we found narratives of stabilization, hybridization, and disruption that show how health literacy concerning COVID-19 is a complex social construct intertwined with emotional, cognitive, and behavioral responses distributed among individuals, communities, and institutions within socioeconomic and political contexts that affect their existence. This paper opens new empirical directions to understand the critical engagement of individuals and communities toward health information aimed at making sense of a complex and prolonged situation of uncertainty in a pandemic.

## Introduction

During the coronavirus disease 2019 (COVID-19) pandemic, diverse stakeholders framed health literacy, in particular digital and media health literacy, as a key public health strategy demanding people to acquire and apply health information to adapt their behaviors at a fast pace (Araújo et al., 2023; Okan et al., 2023; Paakkari and Okan, 2020; Sentell et al., 2020). The traditional approaches to health literacy usually focus on the individual capacities of reading, writing, comprehension, and numeracy skills to make critical informed health-related decisions; communication with physicians; and active participation within modern healthcare systems (Berkman et al., 2010; Samerski, 2019; Sørensen et al., 2012). A variety of empirical quantitative studies have assessed the influence of health literacy on COVID-19 awareness and informational needs, as well as on compliance with preventive behaviors and protective measures, using a traditional approach to health literacy that focuses on individual skills and knowledge (Almusawi et al., 2021; Chen et al., 2022; De Gani et al., 2022; Hermans et al., 2021; McCaffery et al., 2020; Okan et al., 2020; Silva and Santos, 2021). Common topics were the impact of health literacy on mental health (e.g. depression, anxiety, sleeping disorder) (Hermans et al., 2021; Luong et al., 2021; Nguyen et al., 2020, 2021), quality of life and well-being (Ishikawa et al., 2021; Nguyen et al., 2020, 2021); accessibility to health services (Almusawi et al., 2021; McCaffery et al., 2020) and health information (Chen et al., 2022); and trust in the healthcare systems and media information (De Gani et al., 2022; Okan et al., 2020), while others assessed the relationship between health literacy and trust in a range of information sources (Chen et al., 2023).

In the context of the COVID-19 pandemic, health literacy is frequently described as a “weapon” (Araújo et al., 2023), a key to “flattening the curve” (Košir and Sørensen, 2022), and a promising “social vaccine” that can be similarly useful to prevent infection from SARS-CoV-2 as biomedical vaccines (Okan et al., 2023), if applied as a health promotion strategy. Thus, public crisis communication, as well as mass media and public health campaigns, are called upon to educate, persuade and guide people to be “good COVID citizens,” that is, an informed, reflexive and caring person who engages in individual actions on behalf of a unified community (Spoel et al., 2021); to prevent the spreading of “false,” unverified, misleading and contradictory information (Israeli et al., 2022); and to limit the side effects of the COVID-19 “infodemic” (Araújo et al., 2023; De Gani et al., 2022; Naeem and Boulos, 2021; Porat et al., 2020; Sentell et al., 2020). However, the role of social networks and relationships for support with health literacy-related tasks such as information-seeking or decision-making in the context of the COVID-19 pandemic is scarcely understood. In contrast to traditional notions of health literacy, which focus on individual skills and knowledge, we use the concept of distributed health literacy—that is, “the health literacy abilities, skills and practices of others that contribute to an individual’s level of health literacy” (Edwards et al., 2015)—to explore how access and use of health information are shaped by social networks and relationships (Oliffe et al., 2011).

Through the analysis of 89 qualitative interviews with residents from three European countries (Italy, Portugal, and Switzerland) held between October and December 2021, this article moves beyond the traditional approaches to health literacy to explore its social embeddedness (Bauer, 2019; Johnson et al., 2022). Rather than taking health literacy as something that individuals should know and do in a pandemic, we explore how individuals personally perceive the complexity and diversity of skills and resources identified as necessary for people to be considered literate about COVID-19 in their particular socioeconomic and political context, through their relational and lived experiences of the COVID-19 pandemic, and within given structural peculiarities and constraints (Abel and McQueen, 2021; Fox, 2022; Paul et al., 2022; Spahl et al., 2022). In particular, we focus on the role of literacy mediators, that is, “a person who makes his or her literacy skills available to others, on a formal or informal basis, for them to accomplish specific literacy purposes” (Baynham, 1995).

To gain an in-depth understanding of the context and relational processes in the ways people understand specific experiences or phenomena, and, consequently, how they relate their lived experiences to others and social, institutional, and political contexts, we use a narrative-practice approach (Boéri and Giustini, 2023; see also Gubrium and Gubrium, 2021) that aims to overcome the dualism between “action” and “discourse” in traditional social science theory. As proposed by Boéri and Giustini, the narrative-practice approach frames “narrative [as] enacted stories, and practice as storied actions,” and through this strategy interviews in the context of the COVID-19 pandemic function as the practical and narrative site of knowledge construction and contestation that reveals participants’ subjective stories and behaviors against wider meta-discourses (Boéri and Giustini, 2023: 17). Concretely, we explored the dynamic and fluid intersections between “doing” and “saying” distributed health literacy and the complexity of making sense of being “health literate” in people’s lives and worlds. This approach is particularly relevant in the context of a public health crisis such as the COVID-19 pandemic, where behavioral, cognitive, and emotional responses to uncertainty, and the perception of putting oneself and others at risk rely on distributed and situated positionings that are composed of lived experiences in times of crisis (Abel and McQueen, 2021; Fox, 2022; Missel et al., 2021; Spoel et al., 2021). To gain analytical granularity on the participants’ narratives-practices of distributed health literacy in the context of the COVID-19 pandemic, we employed abductive reasoning by critically examining and testing each thematic category developed during the analysis through an iterative cycle of immersion in the data, engagement with relevant theories, interpretation, and formulation of tentative theoretical explanations reviewing the original data in the light of concrete experience (Timmermans and Tavory, 2012; Wagenaar et al., 2022: 9).

## Methods

A total of 89 semi-structured interviews were conducted with members of the general public in Italy (*n* = 24), Portugal (*n* = 38), and the German-speaking part of Switzerland (*n* = 27) between October and December 2021 as part of a qualitative and multinational research study in collaboration with the “Solidarity in times of pandemics” (SolPan) research commons (Wagenaar et al., 2022; Zimmermann et al., 2022b). Due to the ad-hoc setup of the SolPan project, our research design, including the selection of countries, can be best described as pragmatic and opportunistic (Wagenaar et al., 2022). We adopted a flexible strategy in which interpretive analysis was a hermeneutic way of understanding the meaning of similarities and differences of people’s experiences with the COVID-19 pandemic in the three countries by situating them within their relevant context aiming to create the conditions for theoretical innovation, as well as for the possibility to generalize and contextualize findings and explanations (Wagenaar et al., 2022).

Participants were recruited through public advertisements on the university websites, the research groups participating in SolPan, snowballing, and convenience sampling. Most of the interviewees have a high level of education, and medium or high income (Table 1). The inclusion of data from three separate countries afforded greater sensitivity and theoretical saturation regarding the complexity and diversity of skills and resources identified as necessary for people to be considered literate in relation to COVID-19.

**Table 1.** Interviewees’ characteristics.

	Total	Italy	Portugal	Switzerland
	(*n* = 89)	(*n* = 24)	(*n* = 38)	(*n* = 27)
Gender				
Female	50	17	19	14
Male	39	7	19	13
Age				
18–30	16	1	10	5
31–45	24	10	8	6
46–60	23	6	10	7
60+	26	7	10	9
Educational level				
Less than 10 years	11	2	0	9
10–14 years (e.g. high school diploma)	26	12	11	3
Higher education	52	10	27	15
Employment status				
Employed (long-term contract)	28	7	10	11
Self-employed	16	7	6	3
Employed (short-term/precarious contract)	12	1	5	6
Unemployed	6	2	4	0
Retired	19	3	9	7
Other	8	4	4	0
Household composition				
Single	15	5	3	7
Couple	25	7	9	9
Living with child/children under 12	16	5	8	3
Living with child/children 12+	12	3	4	5
Other	21	4	14	3
Urbanity level				
Big town	26	10	11	5
Medium/small town	35	10	16	9
Rural (e.g., village)	28	4	11	13
Household net income				
Up to 1400€ (4000CHF)/month	16	3	8	5
1401€−3000€ (4001–7000CHF)/month	43	15	19	9
More than 3000€ (7000CHF)/month	30	6	11	13

Data was collected based on the SolPan interview guide, where the order and length of the different topics that were covered varied according to the flow of the conversation. The interviews did not focus on health literacy specifically but asked about interviewees’ sources of formal and informal support during the pandemic, as well as the types of skills and resources provided by networks of individuals and communities to deal with COVID-19.

Interviews were held on the phone, online, or in person. Before the interview, participants received in-depth information about the study, and any questions they had were answered. Consent was obtained orally directly before the interview. The consent process and the subsequent interview were recorded on a digital recorder. Audio recordings were stored for transcription. Transcripts were given pseudonyms and not returned to participants.

Throughout the data analysis process, relevant text passages concerning distributed health literacy were selected and analyzed abductively. We searched for distinct narratives that generated a fluid epistemological dialog with practice approaches (Boéri and Giustini, 2023) and aimed to improve theoretical saturation. The coding of each interview was checked by a second researcher for consistency. Through this iterative process characterized by a continual comparison of interviews, we condensed the findings into the three narratives presented in the next section. Following a pragmatic approach (Low, 2019), we addressed data saturation by including data from three European countries, and by aiming to maximize the diversity of perspectives. Quoted participants are anonymous, but each participant is pseudonymized with a country code and a number that are provided after each direct quotation (e.g. “IT10” for Italian participant number 10; “PT12” for Portuguese participant number 12; “CH09” for Swiss participant number 9).

This study received ethics clearance from the University of Vienna (the leading institution of the SolPan research commons, no. 00544), the University of Minho, Portugal (ID: CEICSH 061/2021), and the University of Basel, Switzerland (no. 101).

## Results

According to the narrative-practice approach proposed by Boéri and Giustini, narratives have joint ontological and epistemological mechanisms of construction and appropriation (“patterns of narrativity”) which are the following: temporality (the sequencing of events in time and space), the selective appropriation (selecting or weighting particular events at the expense of others), relationality (the meaning-making connections between the elements of the narrative and between these elements and aspects of the overall context) and causal employment (gearing the plot toward a particular moral outcome) (Boéri and Giustini, 2023: 7). According to the authors, these patterns of narrativity allow people to make sense of who they are (narrative identity), of their relational settings, and how and why the behave as they do in this world (narrative action) (*idem, ibidem*).

The following patterns of narrativity have emerged in data analysis: identity (how interviewees make sense of themselves and others as health literate concerning COVID-19); action (how and why interviewees behave as they do in relation to COVID-19); and relational settings (how and why interviewees engage with or reject literacy mediators for dealing with COVID-19) (Boéri and Giustini, 2023). The following themes unfolded: (1) meanings given to being health literate concerning COVID-19; (2) skills and resources perceived as necessary for people to be considered health literate about COVID-19 (emotional, pragmatic, and informational); and (3) network of literacy mediators for dealing with COVID-19.

Three distinct types of narratives emerged from the data: (1) a narrative of stabilization, when reductionism, similarity, and emotional stabilization are the elements that stand out; (2) a narrative of hybridization, when institutional trust intersects with personal involvement and participation in compliance and education; (3) a narrative of disruption, when humanity and pluralism are perceived as important as medical knowledge and politics to deal with COVID-19.

### Narrative of stabilization

The interviewees that we categorized as enacting a narrative of stabilization were those who mentioned the usual network of health literacy mediators that surrounded them before COVID-19, mainly family members and traditional media (internet, television, and local newspapers). They expressed no particular need to inform themselves proactively about everything.

I still access information in an old-fashioned way via the newspaper and news on TV. And I don’t use any other channels. Because some of it is too lurid for me. I need some information, but I also have to block out certain things. (CH03)

My strongest feeling about this pandemic is that I’ve narrowed [to shape like a funnel], OK? (. . .) I’m less available (. . .) to think about some issues. (. . .) Even today, I choose not to search for information about certain topics. (PT28)

Some participants used to be more proactive in seeking information but stopped at some point because they felt overloaded by the amount of information available, wanted to avoid complexity, inconsistency, and “bad news,” or were just fed up with COVID-19 altogether.

I tried to shield that [media] for myself a little bit. Because (. . .) you hear the left parties, you hear the right parties, and you hear the middle parties. And in the end, you no longer knew what was true and what was not true. (CH18)

The whole [discussion about] “it is best as we do it” and “then we do it that way anyway” and just such things, “we ease [restrictions] there and then we don’t.” At some point I thought, “oh, guys, this [topic] is just getting on my nerves.” And I switched off a bit. (CH22)

As a consequence, several participants stated that they relied on their acquaintances—partners, children, siblings, employers, or friends—to become aware of relevant changes and as information mediators. This was often enough for them to deal with their own health literacy needs, but some interviewees also added “trusted” primary healthcare providers and info hotlines as helpful for managing information related to testing, vaccination, or treatments.

[After I had COVID-19] I started taking vitamins. Only I did it. In my lay opinion, I felt that it would be ideal (. . .) Otherwise, I got advice, especially from my brother, who is a doctor, rather than from the family doctor. Sometimes it’s inconvenient for me to go to the doctor. But I consult with her by phone or by message. As I talk to my brother every day, so I prefer. . . I talk to him. (PT10)

So, at first, I was afraid because (. . .) I thought “If it [the vaccine] has been made so fast there isn’t enough testing and maybe it’s not safe.” Then what convinced me [to get vaccinated] was to ask for medical advice from people. . . from medical doctors whom I trust and whom I have always trusted. (IT01)

The interviewees embracing the narrative that we defined as stabilization frequently mentioned the need to communicate with or receive mediated information from family and friends, with whom they often kept in touch through social media. Participants also referred to the need to be able to select the people they talked to, avoiding discussions with those who disagreed with the handling of the pandemic. Others mentioned that their preferred strategy was adapting information sourcing to the perceived current urgency of the pandemic and obtaining only the minimum relevant information for mental protection.

I have friends (. . .) who on social networks expose theories [on COVID-19] that I do not share. With them, I decided not to discuss this thing. (IT11)

I took myself out of it a bit. I just read our local newspaper (. . .) And from that point of view, I probably don’t know a lot of things that have happened. I never watched any discussion on TV or anything. I don’t need that. (. . .) [It is] a demarcation mechanism and also in the sense of keeping anger away from me when I would get upset. (CH01)

Some interviewees also made their literacy skills available to their loved ones to provide emotional support, while other participants got help registering online for the COVID-19 vaccine. For example, one participant working in the Pharma sector, and another participant working as a family doctor, focused on the provision of emotional support regarding the decision to get vaccinated:

So with people who are really that unsure, who come to me or approach me and ask, “hey, is the vaccination safe or not?” And I tell them “yes” and then they get vaccinated. (CH20)

I am the person they look for to ask for advice [about vaccination]. In general, they trust me. (IT13)

Interviewees undertaking narratives of stabilization commented upon vaccination hesitancy or the belief in conspiracy theories as troubled health literacy skills and practices, sometimes associated with “ignorant” people, as exemplified in the quotes below.

I just find it difficult, this vehemence, when you have the feeling that you’re absolutely on the right side now and everyone else is doing everything wrong. I have a lot of trouble with that. (CH01)

There is a very high percentage of people who are against vaccines in general even before this story. Now to allow these people to spread their ignorance. . .they put people in danger. (IT17)

According to the narratives of these interviewees, being health literate in relation to COVID-19 is not only about self-compliance with rules and recommendations set out by authorities but also about the suppression of self-questioning for the common good.

It was not difficult for me to comply; it was difficult to accept. I always comply, because of this idea that we live in society and that we are citizens and we have to comply with a series of rules, I am OK with that, but I always questioned it. However, I knew that it was for the greater good and I complied. (PT25)

In summary, narratives of reductionism, similarity, and emotional stabilization are important when constructing meaning about how health literacy is distributed throughout a group.

### Narrative of hybridization

For other interviewees, which we categorized as conveying narratives of hybridization, health literacy mediators consisted of a combination of family, friends, and institutional actors linked to the formal settings of science, government, and health authorities. These participants had a very proactive way of seeking health information from a variety of sources, including diverse media formats beyond the daily news (e.g. background reports in the newspaper, talk shows, etc.), and direct information from health authorities and experts. Many also actively engaged in discussions with their social network to exchange information or inform others.

I tried to look at more serious newspapers. Let’s say, more something where I thought they would bring me further in knowledge. Because I think I can only judge or feel one way or the other or talk to other people if I have good, clever arguments. And that’s why I tried to look at the media in a much more targeted way. So maybe not only look at the *Blick* headlines [Swiss tabloid] but also *NZZ* or *Tagesanzeiger* [Swiss quality newspapers]. To read more background reports or to watch talk shows on television. (CH17)

These interviewees often knew how to source and find relevant and trustworthy information, and frequently expressed positive emotions and trust toward scientific and political institutions. Participants valued guidance from politicians and scientists and relied on their recommendations to advise others.

We should try as much as possible to convince that person to be vaccinated, not in a rude way, but by showing facts and research studies so that he/she understands that he/she needs to get the vaccine if he/she wants to help Portugal. (PT07)

To me, it is important to use reputable information sources. That means I am not on Facebook or whatever. (. . .) And also, I trust the Federal Office of Public Health. (CH03)

The role of acquaintances in the provision of emotional support was highlighted as a pillar to deal with anxiety and thus improve mental health, but it is often described as a “private” and “intimate” issue. Some interviewees reported the wish for expertise in their social environment, considered more trustworthy:

It would have been great for me if I knew someone in my circle of acquaintances who, let’s say, had a clue. But I didn’t have that. So the only thing left was the Internet. (CH10)

Besides the provision of informational support to those deemed “uninformed” to allow compliance with action toward the government, the interviewees also provided pragmatic support to the family, friends, and other people to facilitate engagement in protective practices, sometimes upon request or in conjunction with civic and political associations like scouts and parish councils.

Surely information has always been a very difficult issue, in the sense that we have been bombarded with often inconsistent information and therefore it was not easy to identify the official institutional communications and pass them on to the citizens. (. . .) I had activated a whole series of communication channels for the citizens [at the workplace in the public administration], including telephone calls at home. (IT14)

But I’ve also talked to someone who said, “I don’t see why restaurants aren’t open, but the school is open” and so on. And then I try to explain what the difference is between a restaurant and a school and why the conditions are a bit different. (CH05)

I helped my friends to do COVID-19 self-tests because they were afraid to obtain a sample by nasal swab. (PT26)

Initially, when there was a shortage of masks, we, the scouts, distributed food in coordination with the parish council. (PT08)

Health literacy practices situated outside the institutional framework were seen as “irrational.” A few participants perceived highly educated COVID deniers or anti-vaxxers as “ shocking” (CH02) and “surprising” (IT02) as they were expected to be on the side of “truth” because of their educational backgrounds.

The best would not be to force people but to convince them of the goodness of what they are going to do, so . . . I know that sometimes it is not possible because unfortunately there are people who reason by following other logic, not rational ones, and then they think of nonexistent conspiracies. (IT14)

Several participants stressed as crucial elements of health literacy the importance of active information sourcing, the ability to contextualize information, critically assess the trustworthiness of the source, and getting a variety of perspectives.

Even at the beginning of a crisis, you have to have the courage and do the puzzle. Doing the puzzle means using different information channels, and looking at how they communicate this. What is being shown, and what is being talked about? What is the intensity? And to say afterward, based on this puzzle, to form your picture. (CH07)

In summary, according to the interviewees enacting a narrative of hybridization, being health literate in relation to COVID-19 meant complying with rules, following recommendations set out by authorities, and educating other people on how and why to adopt the same path. From the perspective of these participants, institutional trust intersected with personal involvement and participation when constructing meanings about how health literacy was distributed throughout a group.

### Narrative of disruption

The interviewees who searched for information outside the standard network of health literacy mediators did so mainly due to a general distrust of politicians and traditional information sources, particularly the media. We grouped them in what we have named the narrative of disruption. These interviewees criticized the media, including social media, to be tendentious, having conflicts of interest, and reporting overly pessimistically: “they destroy everything” (CH09) and “make terrorism” (IT03). Others accused the media of deliberately ignoring certain aspects of the debate out of economic interests.

The State forces me to get vaccinated, otherwise, I am not allowed to do anything. I became a second-class citizen. (. . .) This is a violation of privacy and most importantly, of democracy. This is very serious. So I am fighting my personal war. Luckily I am not alone. We are many and I hope that consciences will be awakened sooner or later. (IT03)

I wouldn’t know whom I could trust 100% in that sense. I find that it has become such a jungle. You hear one thing and you think, “oh yes, he’s totally right.” And then you hear the opposite somewhere else and find, “oh yes, that’s true.” And then you don’t know anymore, what are the facts now? Yes, because you hear so much about who is sponsored by whom. (CH27)

The following quotation reveals a critical positioning and looking for alternative “humanist” and “scientific” approaches to deal with COVID-19, mainly based on information disseminated by specialists detached from the official mainstream:

I question the ways to control this [COVID-19], there are several, there may be several, and at the moment they [government and media] only impose on us one that is vaccination, and this [imposition] created a great dualism at the social level. (. . .) There were (. . .) people from the health sciences, people who knew what they were saying, who were highly criticized for having a different way of approaching the issue [how to manage COVID-19]. (. . .) In my opinion, the media only adopts this type of narrative, leaving no room for other issues, which are labeled as denialism. (PT16)

Interviewees’ worries about the lack of consistency and the silencing of critical voices in traditional media triggered supporting practices via their social networks. For instance, they provided and received information about complementary or alternative approaches to deal with the diagnosis and treatment of COVID-19. In addition, some of these participants disseminated alternative information they deemed credible, for instance about healthy lifestyles, supported by their examples of resistance, resilience, and happiness. In the words of one interviewee:

I will continue, on my personal page (. . .) and in my personal contacts, (. . .) disclosing information that I find credible. I will try within my workspace to continue to enhance human relationships. (. . .) I show that I am happy, show that I am well, that I am healthy, and that I do not live in fear and do not live subject to measures with which I do not identify. (. . .) This excessive time dedicated to COVID (. . .) contrasts with the lack of information and time dedicated to healthy eating, physical exercise, and human relationships, to demystify fear. (PT14)

Some interviewees also provided pragmatic and emotional support to others by making their health literacy skills available to those they perceived as being vulnerable, namely those who experienced health and economic hardships as a result of the COVID-19 pandemic.

I knew four or five families who needed it [help to eat]. (. . .) I came home to cook, to make the lunch and the dinner to take to them. (. . .) Another lady (. . .) made soup. . . and I made the rest. (PT12)

In summary, for this group of interviewees being health literate concerning COVID-19 means complying with compulsory rules set out by authorities but with critical thought, and being aware of multiple strategies to deal with a pandemic. Values of humanity and pluralism are perceived as important as medical knowledge and politics when constructing meaning about how health literacy is distributed throughout a group.

## Discussion

In line with the theoretical framework of distributed health literacy (Edwards et al., 2015; Muscat et al., 2022), this study conducted in three European countries shows how health literacy in relation to COVID-19 is a complex social construct intertwined with emotional, cognitive, and behavioral responses distributed among individuals, communities, and institutions within situated positionings (Fox, 2022; Spoel et al., 2021). Offering a nuanced picture of the social embeddedness of health literacy (Bauer, 2019), our findings indicate how distinct networks of literacy mediators relate to complex and diverse emotional, pragmatic, and informational literacy skills, as well as to family and social networks identified as necessary for people to be considered literate concerning COVID-19. This study reinforces the importance of context and relational processes to how people understand specific and lived experiences or phenomena in the context of the COVID-19 pandemic (Bröer et al., 2021; Fiske et al., 2022; Galasso and Watts, 2022; Hangel et al., 2022; Johnson et al., 2022; Paul et al., 2022; Spahl et al., 2022), and, consequently, how they rely on others and on social, institutional and political contexts (Gubrium and Gubrium, 2021) to interpret health literacy and their literacy skills available to others in the COVID-19 pandemic.

Our three major distinct narratives show how the participants in this study reproduce, negotiate and contest mainstream rationalizing neo-communitarian governmentalities, discourses and actions on health literacy of “good COVID citizens” (Spoel et al., 2021) through their heterogeneous experiences of the COVID-19 pandemic (Boéri and Giustini, 2023). All the narratives are related to the interviewees’ lives and vital worlds, that is, socioeconomic and political contexts that affect their existence.

The narrative of stabilization is in line with previous studies that indicate that the most common sources of information related to COVID-19 were the internet and traditional media (Ho et al., 2020), with no particular need to proactively inform themselves about everything (Soroya et al., 2021), a strong orientation toward self-compliance with rules and recommendations set out by authorities (Spahl et al., 2022), and subjugation of self-questioning to the common good (Hangel et al., 2022; Zimmermann et al., 2022a). The narrative of hybridization is aligned with previous studies that highlight the role of institutional trust (Badman et al., 2022; Paul et al., 2022) and the personal involvement and participation in compliance with health-related rules set up by official authorities (Spahl et al., 2022). This kind of narrative shows the importance of the use of a hybrid combination of health literacy mediators (family, friends, and institutional actors linked with formal settings from science, government, and health authorities) (Lupton and Lewis, 2021), with a very proactive way of seeking health information from a variety of sources (Ali et al., 2020), and willingness to educating other people (Porat et al., 2020). Finally, the narrative of disruption highlights the importance of humanity and pluralism as important as medical knowledge and politics (Lohse and Bschir, 2020). This latter type of narrative translates into practices of a search for health information outside the standard network of literacy mediators (DiRusso and Stansberry, 2022), mainly due to a general institutional distrust in politicians and traditional information sources (Jennings et al., 2021; van Meurs et al., 2022) and to information gaps related to missing (hidden or silenced) information, manipulated information and discrepant information (Israeli et al., 2022). The group of participants conveying a narrative of disruption referred to engaging in compliance with compulsory rules stipulated by authorities but with criticism, and showing awareness of multiple strategies to deal with a pandemic (Crabu et al., 2023).

In contrast with participants enacting the narrative of stabilization, the participants conveying narratives of hybridization and disruption stated how they were actively contributing to the debate in their social networks and online communities (Lawless et al., 2022). As such, the narratives of hybridization and disruption are related also to “critical health literacy” as a social asset that helps individuals and communities toward a critical engagement with health information (Abel and McQueen, 2021; Chinn, 2011; Oliffe et al., 2011).

To conclude, our findings enrich theoretical and analytical perspectives oriented toward the mapping of the diversity of literacy mediators, showing, for example, how people “protected” themselves by refraining from being exposed to some information either by excluding or avoiding some literacy mediators or selecting others as strategies to deal with information overload and uncertainty and confusion created by divergent opinions and beliefs conveyed by traditional health literacy mediators, like health officials and health communicators (Hodson et al., 2023; Israeli et al., 2022; Soroya et al., 2021). As previous studies about health literacy in the COVID-19 context show, trust shapes the filtering of information sources (De Gani et al., 2022). Our study corroborates the importance of trust but also highlights the relevance of the articulation of trust (and distrust) with emotional, pragmatic, and dynamic informational work aimed at making sense of a complex and prolonged situation of uncertainty.

The findings of this study can offer valuable insights to health communication professionals. Our data emphasize the diversity of sources people turn to for health information during the pandemic. Health communication professionals can use this insight to tailor their messaging and content to align with the various channels and the diverse mediators that individuals utilize. By understanding how people cope with overwhelming information, professionals can design communication materials that help individuals navigate the flood of complex information. Our research also highlights the role of trust in filtering information sources (Chen et al., 2023), and how trust articulates with emotional and pragmatic aspects. Health communication professionals can leverage this insight by focusing on building and maintaining trustworthiness, addressing emotional concerns, acknowledging practical challenges, and providing context for prolonged uncertain situations.

## Limitations of the research

Owing to language and affordability constraints, and because we were covering an array of topics, each author accessed and analyzed only parts of the original data (Wagenaar et al., 2022). However, we have sought to balance this constraint by reporting relevant findings in the English language and jointly discussing the interpretation of the data. Moreover, even though we aimed to enable a maximum variation of views and experiences by controlling key participants’ demographics, our sample is biased toward people with higher levels of education and income. We addressed this issue by following a pragmatic approach of theoretical saturation that applies greater sensitivity to the interpretative process of data analysis derived from three separate countries (Low, 2019).
